# Deciphering spatial microglial diversity in the brain of Alzheimer’s disease patients

**DOI:** 10.1002/alz.088495

**Published:** 2025-01-03

**Authors:** Paula Sanchez‐Molina, Dillon Brownell, Aditya Pratapa, Nadezhda Nikulina, Bassem Ben Cheikh, Jasmine Singh, Alina Bogachuk, Aude Chiot, Gregory Chin, Katie Emberley, Andrea Crotti, Randall Woltjer, Fabian Svara, Oliver Braubach, Bahareh Ajami

**Affiliations:** ^1^ Oregon Health & Science University, Portland, OR USA; ^2^ Akoya Biosciences, Menlo Park, CA USA; ^3^ Vita‐Salute San Raffaele University, Milan Italy; ^4^ NIA‐Layton Oregon Alzheimer’s Disease Research Center, Oregon Health & Science University, Portland, OR USA; ^5^ Ariadne.ai, Lucerne Switzerland

## Abstract

**Background:**

Single‐cell technologies have revealed significant microglial cell heterogeneity across the human brain in both health and disease. However, the integration of high‐plex protein and spatial information in single‐cell approaches constitutes a challenge essential for advancing our cell biology comprehension in the neuroscience field.

**Method:**

In the present study, we employed co‐detection by indexing (CODEX), a protein multiplexed imaging technology, for the first time to unravel the association between different microglial populations and pathological features of Alzheimer’s disease (AD) in the human brain. We used a 32‐plex panel of DNA‐barcoded antibodies able to detect neurons, oligodendrocytes, astrocytes, myeloid cells, vascular components, and pathological markers, in the same brain tissue section. In addition, we implemented algorithms to segment morphologically complex cells and advanced data analysis pipelines.

**Result:**

Our results provide a comprehensive mapping of the human brain cytoarchitecture, showing different cell phenotypes based on their protein expression and morphology, cell interaction dynamics, and cell spatial organizations in healthy and AD individuals. Through the identification of different microglial phenotypes, we found a specific subpopulation associated to amyloid‐ß plaques in AD brains. Interestingly, this subpopulation exhibited shared properties with border‐associated macrophages.

**Conclusion:**

This study presents a novel approach to explore spatial brain cell heterogeneity in the context of neurological diseases and brings new insights into the microglial diversity in AD.

